# Atropine Challenge Test in Screening the Organophosphorus Poisoning Cases with Atypical Presentation; a Brief Report 

**Published:** 2019-08-19

**Authors:** Shahin Shadnia, Nasim Zamani, Sara Nikpour, Ali Saffaei, Mohammad Reza Farnia

**Affiliations:** 1 Department of Clinical Toxicology, Loghman Hakim Hospital, School of Medicine, Shahid Beheshti University of Medical Sciences, Tehran, Iran.; 2 Forensic Medicine Department, Loghman Hospital, Shahid Beheshti University of Medical Sciences, Tehran.; 3 Student Research Committee, Department of Clinical Pharmacy, School of Pharmacy, Shahid Beheshti University of Medical Sciences, Tehran, Iran.; 4 Emergency Department, Imam Reza Hospital, School of Medicine, Kermanshah University of Medical Sciences, Kermanshah, Iran.

**Keywords:** Organophosphorus Compounds, Atropine, Organophosphate Poisoning, Acetylcholine, Toxicity

## Abstract

**Introduction:**

Atropine is not recommended in organophosphorus (OPs) poisoning cases without any obvious clinical signs. This study aimed to evaluate the clinical utility of Atropine challenge test in screening OPs poisoning cases with atypical presentation.

**Methods:**

In this prospective cross sectional study, after primary supportive care, patients with atypical pretentions of OPs poisoning underwent Atropine challenge test (1 mg intravenously) and demographic parameters, clinical presentations, and serum level of cholinesterase enzyme were compared between cases with positive and negative test results.

**Results:**

20 patients with the mean age of 47.60 ± 13.25 years were studied. The mean time since exposure and initial symptoms was 6.17 ± 2.99 hours. The most common clinical presentations were tachycardia (55%) and flushing (35%). The atropine challenge test was positive in 3 (15.00%) cases. The two groups were the same regarding gender distribution (p = 0.582), mean age (p = 0.957), clinical presentation (p > 0.05), and mean PR interval (p = 0.729). The level of cholinesterase was 220.00 ± 15.52 U/mL and 332.17 ± 143.99 U/mL in patients with positive and negative Atropine challenge test, respectively (p = 0.006).

**Conclusion:**

Patients with positive Atropine challenge test had a significantly lower level of serum cholinesterase and response to Atropine in their therapeutic management. Hence, Atropine challenge test could be considered as a useful clinical test in the setting of acute OPs poising.

## Introduction

Organophosphorus (OPs) insecticides are used for agriculture, vector control, and domestic usages. Despite the obvious benefits of these agents, acute OPs poisoning is increasing worldwide (1, 2). Because of their ease of accessibility, OPs products are frequently used for self-poisoning intentions and it is an important public health issue in some developing countries (3, 4). OPs products are the most important source of toxicity and death globally and they cause more than 200,000 deaths every year in some developing countries (5, 6). Acute OPs poisoning can lead to acute cholinergic syndrome, seizures, muscle weakness, loss of consciousness, and respiratory arrest. OPs through the inhibition of acetyl cholinesterase can stimulate both muscarinic, nicotinic, and adrenergic receptors (7). These effects may lead to the accumulation of acetylcholine. Respiratory failure and cardiac arrest are the most usual causes of death in acute OPs poisoning patients (8). Patients with acute OPs poisoning should undergo prompt evaluation and management of disorders in airway, breathing, and blood circulation. Further interventions are based on risk assessment and clinical observations during regular monitoring (9). Once clinical evaluations indicate the use of antidotes, they should be administered promptly. There are three most broadly used classes of antidotes, including muscarinic antagonists (for example: Atropine), oximes (for example: pralidoxime and obidoxime), and benzodiazepines (10). Atropine has no effect on the neuromuscular junction and muscle weakness, therefore oximes are administered to reverse neuromuscular blockage. Atropine is not recommended in patients without any obvious clinical signs (11). However, in cases where the physicians doubt the diagnosis or cases with atypical presentation, Atropine challenge test is recommended (12). The aim of this study was to evaluate the clinical utility of Atropine challenge test in screening the OPs poisoning cases with atypical presentation.

## Methods

***Study design and setting***

In this prospective cross sectional study, patients with OPs poisoning who were referred to the emergency department of Loghman Hakim Hospital, Tehran, Iran, between January 2017 and January 2018, were studied. This center is a national referral center for poisoning and toxicity in Iran. This study was approved by the ethics committee of Shahid Beheshti University of Medical Sciences (ethics code: 19663).

***Participants***

Patients who were between 18 and 65 years old, were exposed to OPs intentionally or accidentally, and had atypical presentation of OPs poisoning (vague or alleviated signs of poisoning and unknown sources of poison) were included. Patients who had received Atropine before presenting to ED or had hepatic or renal failure were excluded. 

***Data gathering***

Age, gender, clinical signs and symptoms (tachycardia, mydriasis, flushing, agitation and dyspnea), PR interval on electrocardiogram (ECG) (lead II and V1), time from exposure to presentation of initial symptoms, and level of serum cholinesterase were recorded for all patients. A toxicology fellowship was responsible for data gathering.

***Atropine challenge test***

After primary supportive care including cardiac monitoring, airway management, supplementary oxygen therapy, and fluid and electrolytes management, Atropine challenge test was performed for all patients. First, basic heart rate of the patient was recorded. Then, Atropine at dose of 1 mg was intravenously (median cubital vein) administered and patient heart rate was monitored. If the heart rate increased more than 20% of its baseline or more than 30 beats per second, the test was considered positive. Other anticholinergic symptoms such as tachycardia, mydriasis, flushing, agitation and dyspnea were recorded. All patients with positive atropine challenge test received atropine as treatment.

***Statistical analysis***

Data were imported into SPSS software version 23.0 (IBM, USA). Findings are presented as mean ± standard deviation or frequency (%). For all the tests (t-test, chi-square), the significance level was considered as 0.05 and results were reported as mean ± standard division (SD) or frequency (%). 

## Results

20 patients with the mean age of 47.60 ± 13.25 years were studied. The mean time since exposure and initial symptoms was 6.17 ± 2.99 hours. The most common clinical presentations were tachycardia (55%) and flushing (35%) (Table 1). On admission, PR interval was 77.95 ± 7.21 milliseconds and mean level of serum cholinesterase was 315.35 ± 138.47 U/mL. Atropine challenge test was positive in 3 (15.00%) cases. Patients with negative Atropine challenge test did not receive Atropine, except one patient. Table 2 compares the baseline characteristics of patients with positive and negative Atropine challenge test. The two groups were the same regarding gender distribution (p = 0.582), mean age (p = 0.957), clinical presentation (p > 0.05), and mean PR interval (p = 0.729). The level of cholinesterase was 220.00 ± 15.52 U/mL and 332.17 ± 143.99 U/mL in patients with positive and negative Atropine challenge test, respectively (p = 0.006).

**Table 1:** Clinical presentations of patients with acute OPs poisoning

**Parameter**	**Frequency (%)**
Tachycardia	11 (55.0)
Mydriasis	5 (25.0)
Flushing	7 (35.0)
Anxiety and Agitation	6 (30.0)
Dyspnea	1 (5.0)

**Table 2:** Comparing the baseline characteristics of patients with positive and negative Atropine challenge test

**Variables**	**Atropine challenge test**		**P Value**
	**Positive**	**Negative**	
**Age (years)**			
Mean ± SD	48.00 ± 12.12	47.53 ± 13.79	0.957
**Gender**			
Male	1 (5.0)	9 (45.0)	0.582
Female	2 (10.0)	8 (40.0)	
**PR interval (millisecond)**			
Mean ± SD	79.33 ± 4.16	77.71 ± 7.70	0.729
**Tachycardia**			
Yes	0 (0.0)	11 (55.0)	0.074
No	3 (15.0)	6 (30.0)	
**Mydriasis**			
Yes	0 (0.0)	5 (25.0)	0.399
No	3 (15.0)	12 (60.0)	
**Flushing**			
Yes	1 (5.0)	6 (30.0)	0.730
No	2 (10.0)	11 (55.0)	
**Anxiety and Agitation**			
Yes	0 (0.0)	6 (30.0)	0.319
No	3 (15.0)	11 (55.0)	
**Dyspnea**			
Yes	0 (0.0)	1 (5.0)	0.850
No	3 (15.0)	16 (80.0)	
**Cholinesterase (U/mL)**			
Mean ± SD	220.00 ± 15.52	332.17 ± 143.99	0.006

Data are presented as mean ± standard deviation (SD) or frequency (%).

## Discussion

Based on the findings of the present study, patients with positive Atropine challenge test had a significantly lower level of serum cholinesterase and response to Atropine in their therapeutic management. Hence, Atropine challenge test could be considered as a useful clinical test in the setting of acute OPs poising. 

Patients with acute OPs poising must undergo prompt evaluation and management (13). Clinical researches in Asia have shown how Atropine can prevent deaths in OPs patients (14). However, the practitioners are still unsure regarding which cases are most likely to benefit from the use of Atropine (15). Since management of these patients should be done promptly, decision making regarding Atropine usage is an important issue. 

The results of the current study showed that Atropine challenge test is a good predictor for necessity of Atropine usage. If the patient referred to emergency department with atypical presentation of OPs poising, the Atropine challenge test can be performed. This way, the initial management strategy can be determined. This test was first introduced in case reports and based on our knowledge there is not any systematic study in this regard. In one study, which was done by Cappato et al., the clinical applicability of Atropine challenge test was evaluated in discriminating organic from autonomic involvement of sinus automaticity (16). They found that atropine test is not very helpful in discriminating between an organic and an autonomic involvement of sinus automaticity in patients with sinus bradycardia. Another point about Atropine challenge test was discussed by Erdman et al. They previously noted that Atropine challenge test has never been empirically tested and may not be very sensitive or specific (17). 

It seems that patients with positive Atropine challenge test required Atropine in their therapeutic management and those with negative Atropine challenge test may not require Atropine. Hence, Atropine challenge test may be considered as Atropine requirement indicator, and it is recommended to evaluate every patient with atypical presentations of OPs poisoning with Atropine challenge test.

## Limitations

The main limitation of current research was its small sample size, however this was due to low incidence of OPs poisoning with atypical presentations. The advantage of current research was its novelty, which introduced Atropine challenge test as a crucial diagnostic test. 

## Conclusion:

Patients with positive Atropine challenge test had a significantly lower level of serum cholinesterase and response to Atropine in their therapeutic management. Hence, Atropine challenge test could be considered as a useful clinical test in the setting of acute OPs poising. 
